# Arbuscular Mycorrhizal Fungal Inoculation Increases Organic Selenium Accumulation in Soybean (*Glycine max* (Linn.) Merr.) Growing in Selenite-Spiked Soils

**DOI:** 10.3390/toxics10100565

**Published:** 2022-09-26

**Authors:** Zengyu Zhang, Bei Li, Yongxian Liu, Lixin He, Ting Pang, Zongdao Chen, Md. Jahidul Islam Shohag, Xiuyan Miao, Xi Li, Minghua Gu, Yanyan Wei

**Affiliations:** 1 Cultivation Base of Guangxi Key Laboratory for Agro-Environment and Agro-Products Safety, State Key Laboratory for Conservation and Utilization of Subtropical Agri-Bioresources, College of Agriculture, Guangxi University, Nanning 530004, China; 2 Guangxi Academy of Agricultural Sciences, Nanning 530004, China; 3 Soil and Fertilizer Workstation of Guangxi Zhuang Autonomous Region, Nanning 530004, China; 4 Agricultural Service Center of Guangxi Liubei District, Liuzhou 545000, China; 5 Department of Agriculture, Bangabandhu Sheikh Mujibur Rahman Science and Technology University, Gopalganj 8100, Bangladesh; 6 College of Agriculture and Food Engineering, Baise Uninversity, Baise 533000, China

**Keywords:** selenium, arbuscular mycorrhizal fungi, soybean

## Abstract

Selenium (Se) is an essential trace element for humans. Arbuscular mycorrhizal fungi (AMF) play a crucial role in increasing plant micronutrient acquisition. Soybean (*Glycine max* (Linn.) Merr.) is a staple food for most people around the world and a source of Se. Therefore, it is necessary to study the mechanism of Se intake in soybean under the influence of AMF. In this study, the effects of fertilization with selenite and inoculation with different AMF strains (*Claroideoglomus etunicatum* (Ce), *Funneliformis mosseae* (Fm)) on the accumulation and speciation of Se in common soybean plants were discussed. We carried out a pot experiment at the soil for 90 days to investigate the impact of fertilization with selenite and inoculation with Ce and Fm on the Se fractions in soil, soybean biomass, accumulation and speciation of Se in common soybean plants. The daily dietary intake of the Se (DDI) formula was used to estimate the risk threshold of human intake of Se from soybean seeds. The results showed that combined use of both AMF and Se fertilizer could boost total Se and organic Se amounts in soyabean seeds than that of single Se application and that it could increase the proportion of available Se in soil. Soybean inoculated with Fm and grown in soil fertilized with selenite had the highest organic Se. The results suggest that AMF inoculation could promote root growth, more soil water-soluble Se and higher Se uptake. The maximum Se intake of soybean for adults was 93.15 μg/d when treated with Se fertilizer and Fm, which satisfies the needs of Se intake recommended by the WHO. Combined use of AMF inoculation and Se fertilizer increases the bioavailable Se in soil and promotes the total Se concentration and organic Se accumulation in soybean. In conclusion, AMF inoculation combined with Se fertilization can be a promising strategy for Se biofortification in soybean.

## 1. Introduction

Selenium (Se) is an essential trace element for humans that is necessary for the biosynthesis of antioxidant enzymes such as glutathione peroxides [1,2]. Se deficiency in human nutrition can cause a variety of health problems and increased risk of many kinds of human pathologies, including colorectal adenoma and cancer [3]. Several studies have reported Se-deficient soils in more than 40 countries, especially in some parts of China [4,5,6]. In China, it is estimated that almost 2.5 million people suffer from various diseases caused by Se insufficiency [7]. Intake of foods with inadequate Se, such as cereals, is the main cause of global Se deficiency in humans [8]. Cereals, such as soybean (*Glycine max* (Linn.) Merr.), are some of the most common foods in the human diet around the world; hence, they are considered to be the primary source of Se for humans [9]. Since Se in the soil can enter the food chain through plant uptake, plants provide Se to the remainder of the food chain [10]. As a result, levels of Se in soybeans, particularly in their seeds, must be controlled to provide adequate human consumption.

Biofortification, an agronomic technique, and Se fertilization are both cost-effective and effective strategies for increasing Se levels and bioaccessibility in plants [11]. The amount of Se in plants is strongly influenced by the concentration of Se in soil. Se fertilizer species affect the mobility of Se in the soil and its availability to plants. Inorganic Se fertilizers, such as selenite (SeIV) or selenate (SeVI), have been successfully utilized to enrich Se in agricultural products, which was considered as the most effective Se-fertilization approach for various arable cereals [12]. However, a dramatic increase in Se application in agricultural soil will ultimately pose a potential risk to the environment, food safety, and human health [13]. Therefore, the use of Se fertilizer on crops in a suitable manner is an efficient and safe agricultural approach that aims to produce Se-enriched products to supplement dietary Se [14,15].

Plant development and micronutrient acquisition can be boosted by a variety of microorganisms [16]. Arbuscular mycorrhizal fungi (AMF) are common in soil and can create symbiotic relationships with the roots of nearly all plant species, resulting in improved plant performance and soil quality [17]. The AMF allows for an alternate pathway of nutrient assimilation through the interface of the extra-radical and intracellular hyphae, obstructions, and root apoplast [18]. According to prior studies, the most prominent feature of AMF’s range of services is the uptake of immobile nutrients including P, Zn, Fe, Ca [19,20]. Only a few studies have investigated the effects of AMF inoculation on plant Se absorption. However, most of them have demonstrated that AMF is an effective method for increasing Se concentration in food crops (i.e., winter wheat, rice, garlic and onion) [21,22,23,24,25] The effects of AMF on soybean growth and the accumulation of Se in soybean seeds has received little attention thus far.

Se speciation, in addition to the overall Se concentration, is another important factor affecting the health benefits of various plant edibles. Se speciation has been accomplished in a wide variety of plants, ranging from Se-accumulators to cereal grains and vegetables [26], but it has yet to be completely researched or understood in soybeans, one of the most important and commonly consumed crops. It is well known that the major Se species in plants are SeIV, SeVI, selenocysteine (SeCys), and selenomethionine (SeMet) [27]. Organic forms of Se were found to be more bioavailable than inorganic forms and to be more effective in increasing blood Se concentrations (suggesting better absorption and retention) in previous research [28]. Therefore, both organic Se and its concentration should be taken into account when evaluating the biofortification of plants with Se.

Thus, the objectives of this study are: (i) to assess the effect of different AMF and single Se fertilizations on concentration and species of Se in soybean seed; (ii) to investigate whether or not AMF inoculation and Se fertilization can alter soil Se fraction, which could influence Se uptake by soybean; and (iii) to determine the dietary Se intake risk of soybean after AMF inoculation and Se fertilization, as well as use daily dietary intake (DDI) to determine whether or not soybean can meet the daily Se intake by humans. These findings can be used to construct a trial to assess the feasibility of AMF inoculation and Se fertilization in increasing Se accumulation as a viable technique for Se biofortification programs in the future.

## 2. Materials and Methods

### 2.1. Pot Experiment

Soybean (*Glycine max* (Linn.) Merr. Meilifengmao NO. 2) seeds were purchased from the Oil Crops Research Institute, Chinese Academy of Agricultural Science, Wuhan, China. Soybean seeds were surface-sterilized with 70% ethanol for 1 min and 0.01 g/mL sodium hypochlorite for 5 min, thoroughly rinsed with deionized water, and germinated for 48 h at a humidity of 85% and a temperature of 37 °C in the dark for subsequent experiments.

A pot experiment was conducted at Guangxi University in Guangxi, China. The soil used for the pot experiments was taken at a depth of 0–20 cm from an agricultural area in Qinzhou, Guangxi, China. The physicochemical parameters of the soil samples were as follows: pH of 5.8, alkaline hydrolytic nitrogen 210.43 mg/kg, available phosphorus 52.19 mg/kg, available potassium 22.70 mg/kg and total Se content 0.46 mg/kg. Soils were air-dried and sieved at a size of 2 mm. The soil was autoclaved (121 °C for 120 min) for subsequent experiments. At the start of the pot experiment, basic N, P, and K fertilizers were supplemented in each pot as urea, calcium phosphate, and potassium sulfate (1.63, 0.96, and 0.78 g), respectively. The experiment was designed following a randomized block design using a factorial combination of two Se treatments and three mycorrhizal inoculations. The two Se treatments included control (untreated sample) and 0.50 mg of Se, in the form of Na_2_SeO_3_, per kg of soil. Na_2_SeO_3_ is dissolved in 100 mL of water and sprayed into the soil as a spray using a watering can. The three mycorrhizal inoculations included control (non-mycorrhizal treatment) and inoculations with two different AMF strains (*Funneliformis mosseae* (Fm) and *Claroideoglomus etunicatum* (Ce)), which were provided by the Beijing Academy of Agricultural and Forestry Science, Beijing, China. For mycorrhizal treatment, each pot received a combination of 5 kg soil and 200 g inoculum, then were blended. The same soil and sterilized inoculum size ratio were used for controls (non-mycorrhizal treatment), and each treatment was carried out in triplicate. In each pot, five pre-mined soybean seeds were sowed. The bean seedlings were reduced to three plants per pot after germination. The plants were cultivated at temperatures of 20 °C/16 °C day/night and 14 h/10 h day/night. During the plant growth period, the soil moisture was maintained at 70% of its water-holding capacity. The soybean plants were harvested after 90 days and carefully washed with tap water, then deionized water. Seeds, pods, leaves, stems, and roots were separated from the plant samples.

### 2.2. Measurement of Plant Dry Weights (DWs) and AMF Mycorrhizal Colonization in Roots

The whole mature plant, including the root system, was harvested after 90 days. Fresh plant samples were thoroughly washed with distilled water. To acquire the DWs of individual organs, all samples were dried in an oven for three days at 80 °C. Each soybean tissue was weighed using a 1/1000 scale, and the DWs data of each soybean part were recorded. The dried seeds, leaves, stems and roots were crushed separately, passed through 2 mm sieve, and stored in ziplocked bags for subsequent determination of Se concentration and Se speciation of soybean seeds. After soybean harvest, soil samples from each treatment were collected separately and air-dried, hammered, and sieved before being stored in ziplock bags for subsequent analysis of soil Se fraction.

The colonization of mycorrhizal fungi was measured using roots. The roots were cut into 1.0 cm long fragments that had previously been reserved. Root segments were cleared in 10% potassium hydroxide in a water bath for 10 min at 90 °C, washed with water, and then stained with 0.1% trypan blue for 3–5 min at 90 °C in a water bath. The infected roots were loaded into slides, and the AMF colonization of soybean roots was observed under a microscope, and the colonization rate of soybean roots was determined by the method of Li et al. [29].

### 2.3. Determination of Soil Se Fractions

Soil Se fractions in each sample were analyzed using the five-step sequential extraction method reported by Wang et al. [30]. Briefly, (1) water-soluble Se (SOL-Se): in a 100 mL polyethylene plastic centrifuge tube, 1.000 g soil was accurately weighed and 10 mL of 0.25 mol/L KCl was added. The mixture was then centrifuged at 4000 rpm for 15 min after being shaken at room temperature for 1 h. The supernatant was kept at 4 °C for SOL-Se analysis. (2) Exchangeable Se and carbonate-bound Se (EXC-Se): the precipitate from step (1) was added with 10 mL of 0.7 mol/L KH_2_PO_4_ (pH = 5.0) and extracted for 4 h at 25 °C, with the supernatant collected for EXC-Se analysis. (3) Fe-Mn oxide-bound Se (FMO-Se): in step (2), 10 mL of 2.5 mol/L HCl was added to the precipitate, and the supernatant was collected for FMO-Se analysis after an hour of intermittent oscillatory extraction at 90 °C. (4) Organic-bound Se (OM-Se): for organic matter-associated Se analysis, 8 mL of 5% K_2_S_2_O_8_ and 2 mL (1:1) HNO_3_ were added to the precipitate in step (3) and subjected to intermittent shaking extraction at 90 °C for 3 h. (5) Residual Se (RES-Se): the residual precipitate was treated with a 4:1(*v*/*v*) mixture of HNO_3_ and HClO_4_, and the mixture was digested at 160–170 °C. An inductively coupled plasma mass spectrometer (ICP-MS) was used to quantify the concentrations of Se in the extraction processes.

### 2.4. Determination of Se Concentration in Soybean

According to a previous study, the plant Se concentration was tested using the method described by Sha et al. [31]. Briefly, 0.2000 g plant samples were digested with 8 mL HNO_3_ in a tube at 180 °C in a DigiPREP apparatus (MARS 6, CEM, Matthews, NC, USA) for 2.5 h. After that, as an oxidant, 0.5 mL hydrogen peroxide was added twice (30 min interval). The tubes were filled with 15 mL of 2% HNO_3_ after cooling to room temperature, and the concentration of Se in the digester was analyzed using ICP-MS.

### 2.5. Analysis of Se Speciation in Soybean Seed

The analysis of Se fractions in seeds was conducted as described by Dai et al. [27]. using HPLC-ICP-MS following ultrasound-assisted enzymatic extractions with protease and α-amylase. Separation of different Se species was carried out using a Hamilton PRPX100, 250 × 4 mm, column. The ICP-MS peaks at *m*/*z* 78 and 80 were monitored, and the flow rate of hydrogen flowing into the octopole reaction cell was 4 mL/min. The injection volume was 50 μL per sample. NH_4_NO_3_ (40 mM) was used as the mobile phase. In brief, 0.2000 g of freeze-dried powdered plant samples was extracted with 10 mL deionized water for 30 min under sonication, centrifuged for 10 min, and filtered through a filter paper and then through a 0.45 μm filter. The powdered samples were then mixed with 5 mL of ultrasonic extraction solution and centrifuged at 5000× *g* for 20 min. For the Se speciation analysis, the samples were filtered through a 0.22 μm membrane and kept at 4 °C. Na_2_SeO_3_, Na_2_SeO_4_, selenocystine, selenomethionine, and semethyl-selenocysteine were used to make stock solutions of SeIV, SeVI, SeCys, SeMet, and MetSeCys. Quantifications of samples were carried out based on peak areas using an external calibration curve or by using the standard addition method, depending on the sample dilution. The recovery rate of SeIV, SeVI, SeCys, SeMet, and MetSeCys ranged from 80% to 110%.

### 2.6. Calculation of Bioaccumulation Factors (BAFs) and Translocation Coefficients (TCs)

In order to explore the ability of soybean plants to absorb Se under the influence of AMF inoculation, bioaccumulation factors (BAFs) and translocation coefficients (TCs) were used to represent the ability of soybean roots to absorb Se and the transport of Se in soybean plants. BAF is the ratio of Se concentration in soybean root to the total Se concentration in soil, and TCs are the ratios of Se concentrations in roots to shoots samples (stems, leaves, pods, seeds). The calculation formulas are as follows:

BAFs = C_root_/C_soil_

TCs = C_i_/C_root_

where C_soil_ is the total Se concentration in the soils, C_root_ (mg/kg) is the concentration of Se in the corresponding roots, and C_i_ (mg/kg) is the concentrations of Se in soybean organs [32].

### 2.7. Se Biofortification Potentiality Analysis

For different groups of people, the World Health Organization (WHO) suggested a daily limit of 55 to 200 µg Se/day [33]. The toxic daily intake of Se for an adult is 400 μg/day [34]. As a result, the daily intake of Se from soybean seeds for humans was calculated in this study. The following equation was used to calculate the daily dietary intake (DDI) [13].

DDI(μg) = C_Se_ × D_food intake_ × 1000

C_Se_ and D_food intake_ represent Se concentration in the seeds of soybean (mg/kg) and daily intake of soybean (kg), respectively. The daily intake of vegetables (soybean) for an adult is considered to be 0.345 kg/person/d.

### 2.8. Statistical Analysis

The average of three replications was used to analyze all of the findings. The Duncan’s Test was used to compare treatment differences at a 5% level of significance. The SPSS 20.0 software was used to perform one-way ANOVA and two-way ANOVA analysis of variance.

## 3. Results

### 3.1. Root Colonization and Plant Development

No mycorrhizal colonization was observed in soybean roots of the uninoculated sample. The roots of inoculated soybean were extensively colonized (30.14–34.13% of root length colonized) by AMF (Table 1). Se fertilization had no significant effect on soybean growth and grain yield (*p* > 0.05) (Table 1). Mycorrhizal inoculation significantly influenced the biomass of soybean individual organs (*p* ≤ 0.05) (Table 1). Regardless of Se fertilizer application, mycorrhizal inoculation increased soybean biomass compared with non-inoculation (control) treatments. However, the leaf and stem dry weight increased significantly in mycorrhizal plants only in Se-spiked soils.

### 3.2. Fractions of Se in Soil

**Figure 1:**
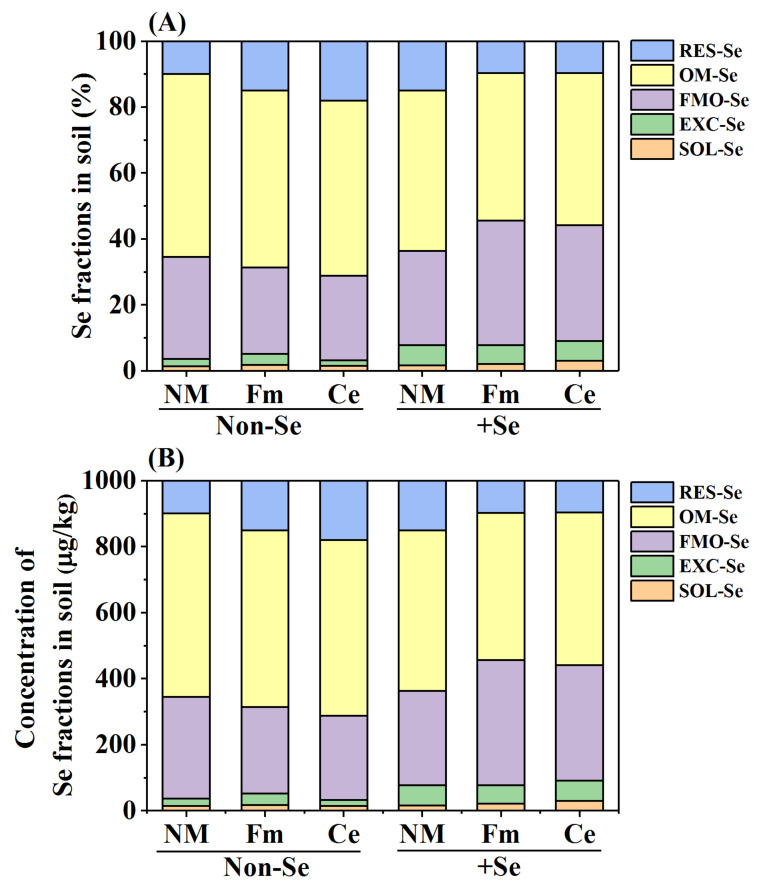
Effects of AMF inoculation and Se fertilization on distribution of Se fractions (**A**) and the concentration of Se fraction (**B**) in the soil. Here, SOL-Se indicates water-soluble Se, EXC-Se indicates exchangeable and carbonate-bound Se; FMO-Se indicates Fe/Mn oxide-bound Se; OM-Se indicates organic matter-bound Se; and REC-Se indicates residual Se. NM indicates the treatment without AMF, Ce indicates *Claroideoglomus etunicatum*; Fm indicates *Funneliformis mosseae*.

Se fertilization had a significant impact on the distributions of Se in different fractions (*p* ≤ 0.05) (Figure 1A), including SOL-Se, EXC-Se, FMO-Se, OM-Se and RES-Se. In the non-Se treatment, the Se concentration of SOL-Se ranged from 13.93 to 17.42 µg/kg, EXC-Se Se ranged from 17.46 to 33.96 µg/kg, FMO-Se Se ranged from 256.24 to 308.93 µg/kg, OM-Se Se ranged from 531.37 to 555.41 µg/kg, and RES-Se Se ranged from 99.56 to 180.49 µg/kg (Figure 1B). In the Se treatment, the Se concentration of SOL-Se ranged from 15.82 to 29.95 µg/kg, EXC-Se Se ranged from 56.75 to 61.71 µg/kg, FMO-Se Se ranged from 285.55 to 378.07 µg/kg, OM-Se Se ranged from 446.92 to 486.20 µg/kg, and RES-Se Se ranged from 97.03 to 150.72 µg/kg (Figure 1B). Regardless of Se fertilizer application, OM-Se is the dominant fraction, accounting for 44.69–55.54% of total Se concentration in soil. SOL-Se concentration is the lowest at 1.39 to 3.00% of the total soil Se concentration.

Furthermore, AMF inoculation had a significant effect on the proportions of the Se fractions in Se-spiked soil, which varied depending on the AMF strains (Figure 1). Compared with non-mycorrhizal treatment, Fm inoculation significantly increased the SOL-Se, EXC-Se, FMO-Se, and OM-Se proportion with a value of 63.68%, 14.03%, 64.15%, and 13.97%, respectively, while decreasing the RES-Se fraction with a value of 19.90%. Additionally, Ce inoculation significantly increased the fractions of SOL-Se, EXC-Se, FMO-Se, and OM-Se fractions by 140.57%, 24.67%, 56.05%, and 20.64%, respectively, while decreasing the RES-Se fraction by 18.22%.

### 3.3. Distribution of Se Concentration in Soybean Organs

**Figure 2:**
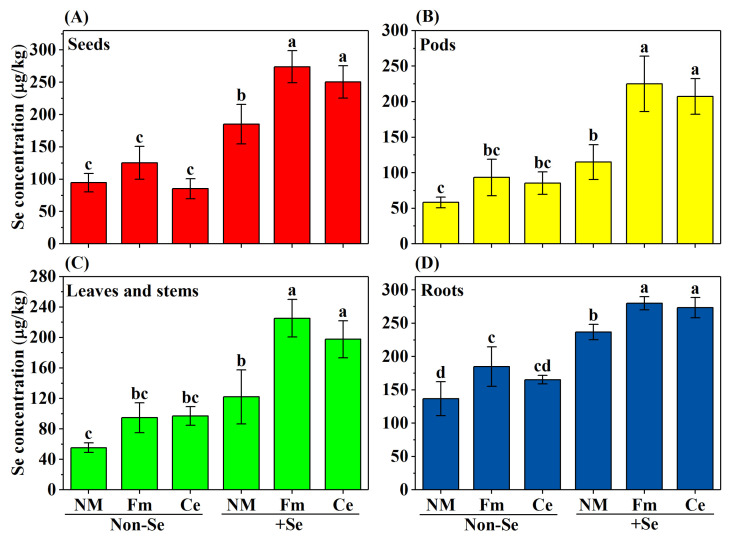
Effects of AMF inoculation and Se fertilizations on Se concentration in soybean organs at full maturity. (**A**) Seeds, (**B**) pods, (**C**) stems and leaves, (**D**) roots. NM indicates the treatment without AMF, Ce indicates *Claroideoglomus etunicatum*, Fm indicates *Funneliformis mosseae*. Within each column, data presented are the means ± SE of three independent pot replicates. Data with different letters reveal significant differences in the same color column (*p* ≤ 0.05).

The effects of both the AMF inoculation and Se fertilizer on the Se concentration in plants are shown in Figure 2 and Table 2. AMF inoculation significantly increased the Se concentration in soybean organs with Se fertilization compared to the non-Se application. Compared with the non-inoculation in Se fertilizer treatment, soybean inoculated with Fm and Ce significantly increased the Se concentration by 0.42 and 0.32 times in seeds (*p* ≤ 0.05); inoculated with Fm and Ce significantly increased the total Se concentration by 0.92 and 0.75 times in pods (*p* ≤ 0.05); in stems and leaves, Se concentration increased by 0.92 and 0.67 times (*p* ≤ 0.05); in roots, Se concentration increased by 0.17 and 0.13 times (*p* ≤ 0.05).

### 3.4. Speciation of Se in Soybean Seed

**Figure 3:**
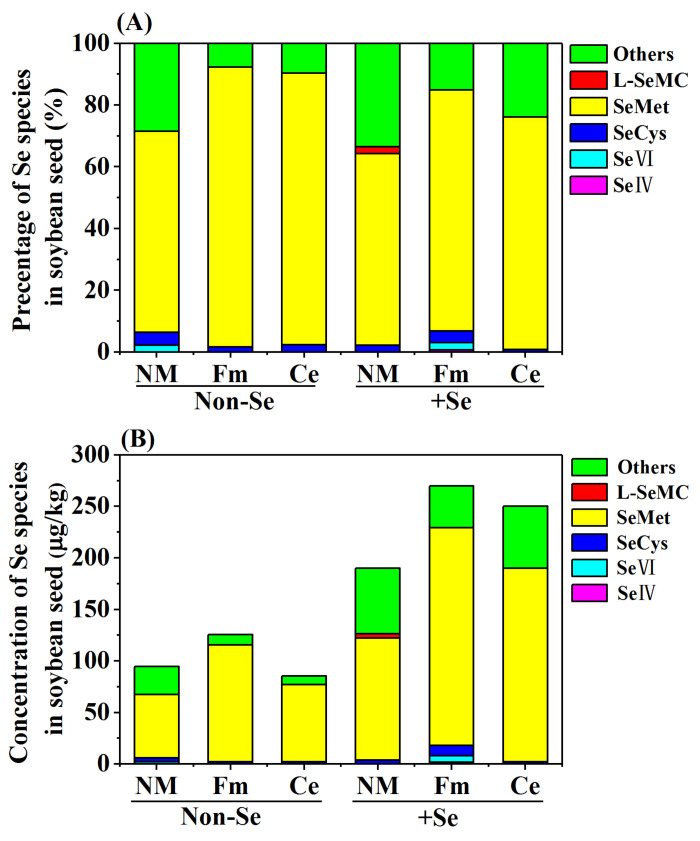
Effects of AMF inoculation and Se fertilizations on Se species (**A**) and the concentration of Se species (**B**) in soybean seed.

AMF inoculation and Se fertilization had significant effects on Se speciation in soybean seed (Figure 3A). Both inorganic and organic Se species (SeIV, SeVI, SeCys, SeMet, and L-SeMC) were observed in soybean seed, which varied with different treatments. In the non-Se treatment, SeCys and SeMet were mainly detected, and the concentration of SeCys ranged from 2.04 to 4.07 µg/kg, SeMet ranged from 61.50 to 113.50 µg/kg (Figure 3B). In the Se treatment, SeCys and SeMet were also the main Se species, and the concentration of SeCys ranged from 2.04 to 10.18 µg/kg, and SeMet ranged from 118 to 211 µg/kg (Figure 3B). According to this study, SeMet accounted for 62.11% to 90.56% of the total Se concentration and was the dominant Se species detected in soybean seeds in all treatments. The application of selenite increased the fraction of SeMet by 85.09% to 150.67% compared to the non-Se treatment. Moreover, Fm and Ce inoculations can increase the proportion of SeMet in soybean seed by 78.81% and 59.32%, respectively, compared to a single application of Se.

### 3.5. Bioaccumulation Factor and Translocation Coefficients of Se in Soybean

To reflect the ability of soybeans to absorb Se from the soil, the BCFs in roots for AMF inoculation and Se fertilization were calculated (Table 3). Compared with non-Se fertilizations, Se fertilization could significantly increase the BAF of Se from soil to root. Additionally, BAF was significantly increased in the root of soybean inoculated with Fm and Ce (*p* ≤ 0.05).

To reflect transport of Se in plants, translocation of Se from root to straw, from straw to pods, and from pods to seeds for AMF inoculation and Se fertilization were calculated (Table 3). For the translocation of Se from root to aboveground portions of soybean, the TCs of Se were higher in soybean grown in the present study. Moreover, AMF inoculation and Se fertilization had a significantly positive impact on the translocation of Se from root to the aboveground portion (*p* ≤ 0.05). Inoculated with Fm had the highest TCs of Se in the seed when Se fertilizer was applied.

### 3.6. Se Biofortification Potentiality Analysis

Estimated daily Se intake levels are shown in Table 4. For adults, the daily intake of Se from soybean treated with Se fertilizer or Se + AMF was estimated to be 32.66–93.15 μg (Table 4). The maximum selenium intake of adults was 93.15 μg when treated with Se fertilizer and Fm, which reached the Se intake recommended by the WHO. As a result, the daily intake of Se from soybeans from plants inoculated with AMF and Se-fertilized soil has not been observed to be toxic to humans. Moreover, soybeans grown in the soils treated with Se and inoculated with Fm had the highest daily intake of Se by adults but did not exceed the toxicity level of 200 μg/day.

## 4. Discussion

Mycorrhizal symbiosis plays a critical role in plant viability [35]. The present study revealed that soybean plants grown using AMF could significantly increase the colonization rates of roots (30.14–34.14%) and the dry weight of soybean organs compared to non-inoculation treatment (Table 1). The results are consistent with previous reports that root colonization and soybean organs dry mass in mycorrhizal treatment were significantly higher than that in non-mycorrhizal plants [36,37]. However, the mycorrhizal infection that occurred in soybean roots inoculated with different fungi indicated that mycorrhizal symbiosis was still active in mature soybean roots, regardless of Se fertilization. Therefore, in this study, both Ce and Fm inoculation increased soybean dry weight, indicating that AMF mycorrhizal inoculation promoted soybean plant growth. Adeyemi observed similar results in his study, which found that using the commercial AMF inoculant could improve soybean growth and yield under favorable conditions [38]. This is because the roots of plants infected with AMF can more efficiently utilize phosphorus and nitrogen in the soil, thus promoting the growth and development of plants and increasing the biomass [39]. Therefore, in this study, soybean inoculated with AMF increased the uptake of nutrients by increasing its root colonization rate, which promoted growth and increased soybean yield.

The fractionation and speciation of Se in the environment are important biogeochemical indicators of soil biogeochemical properties such as Se mobility and bioavailability and Se uptake by plants [40]. It is generally believed that SOL-Se and EXC-Se fractions are readily available to plants, and therefore, SOL-Se and EXC-Se are used to determine the bioavailability of Se in soil [34]. Se bioavailability in the soil is influenced by a variety of factors, including Se sorption or/and desorption from the soil [40]. In the present study, we noted that Se fertilization and AMF inoculation could modify the pattern of Se distribution in the soil. In contrast to the non-Se treatment, Se fertilization increased SOL-Se and EXC-Se in the bean-grown soil, with OM-Se being the dominant fraction regardless of Se fertilization. The results of the non-Se fertilization treatment are consistent with those of a prior study, which has reported that OM-Se mainly exists in the soil without Se addition soils, in which it reaches 44.49–46.47% of the total Se [25]. Therefore, mycorrhizal inoculation in this study not only increased the biomass of soybean, but also increased the contents of SOL-Se and EXC-Se in soil, thus promoting the absorption of Se in soybean. OM-Se was found to be the main Se fraction in SeIV-spiked soil in our study, which was different from the results of previous studies [25]. This result may be due to the lower availability of SeIV, as it is more easily fixed by soil particles. The available Se (the contents of soluble and exchangeable Se fractions) in soil rapidly decreases at the initial stage of aging (30 d) and slowly declines thereafter during aging. Hence, the Se fraction can shift from highly available Se to organic matter-bound (OM-Se) with aging [30,41,42]. Therefore, we are more certain that SOL-Se and EXC-Se are the most readily available Se fraction for plants, regardless of the variation of Se fractions in the soil. Therefore, in this study, AMF inoculation and Se fertilizer application promoted the transformation of SOL-Se and EXC-Se in soil, making Se into a form more conducive to soybean absorption. In addition, we found in this study that AMF inoculation has a significant impact on the proportions of Se fractions in the soil, which varied depending on the AMF strains. Especially, the inoculation with Fm significantly increased the SOL-Se and EXC-Se fractions in soil, respectively, stating that inoculation with Fm significantly increases the proportion of available Se.

Crops have markedly different capacities for Se uptake. The crop absorbed Se from the soil via upward translocation in the root, then distributed Se to different tissues, and ultimately deposited Se in the grain (as reflected by grain Se accumulation). Numerous studies have found that increasing Se concentration in the soil using inorganic Se fertilization has positive impacts on Se accumulation in several crops, including Zea mays, soybean, and wheat [21,43,44]. In the current study, we found that fertilizing the soil with selenite could significantly increase the concentration in different parts of soybeans, including roots, leaves and stems, pods, and seeds. In the different Se fertilization treatments, the concentration of Se was higher in the soybean seed than in the rest of the plant. The results indicated that selenite had optimal promotion effects at 0.5 mg Se/kg dry soil, which could best boost Se accumulation in soybean seed. This funding is consistent with a previous study [45]. Combined with the results of soil Se fractions in this study, it was shown that exogenous Se application could change soil Se fractions and make soil Se more easily uptaken by plants. Moreover, the inoculation with AMF resulted in a significant increase in the concentration of Se in different parts of the soybean. Fm inoculation was found to promote better absorption of Se by soybean plants better than the Ce inoculation. Mycorrhizal inoculation has been shown to accelerate Se uptake in host plants and has been observed in many plants, such as garlic and onions [24]. In conclusion, exogenous Se application and AMF mycorrhizal inoculation promoted Se uptake and utilization in soybean by changing the soil Se fraction and increasing plant root area.

BAF is a measure of a plant’s ability to accumulate trace elements from the soil environment [46]. Our findings revealed that during Fm inoculant treatment, the highest BCFs of Se in soybean root reached 0.56 (Table 3), reflecting the ability of soybeans to absorb Se. TC is used to reflect the transport of elements or ions in plants [45]. Our study found that the TCs of Se in seeds are much higher than in other organs and are significantly affected by mycorrhizal inoculation and Se fertilization. The changes in BAFs and TCs indicated that the combine used of AMF and Se fertilizer promoted the uptake of Se in soybean roots and affected the transfer of Se in soybean organs, which was beneficial to the conversion of inorganic Se to organic Se in plants. SeIV is thought to be rapidly converted into organic selenium compounds in the roots of plants, and to be subsequently transferred to the plant’s aboveground portions [47]. Organic selenium compounds may therefore be produced to a greater extent with AMF treatment; however, this mechanism requires further verification and exploration.

Se speciation in edible plant parts is an important biofortification factor for human health. According to one study, organic Se is superior to inorganic Se in terms of bioavailability, safety and anti-cancer effects [28]. In the present study, SeMet was the major Se species observed in the soybean seed, regardless of AMF treatment. This finding is consistent with the results of studies on cereals such as wheat [48] and rice [49]. However, in the present study, inoculation with AMF improved the accumulation of SeMet in soybean seed more effectively compared to non-Se fertilization. The proportion of SeMet in soybean was increased by Fm and Ce inoculation. This result suggests that the inoculation with AMF may be more effective in improving the accumulation of SeMet in soybean seeds. The result is consistent with Yu et al. [50], who has observed that mycorrhizal inoculation increased the organic Se proportion (including SeMet) in alfalfa. Therefore, our results showed that AMF inoculation and exogenous Se application could change soil Se fractions, promote Se uptake by soybean, and increase organic Se accumulation by soybean compared with single inorganic Se fertilizer treatment.

Finally, in order to test the safety threshold of Se concentration of soybean produced under the combined use of AMF and selenium fertilizer in this study for human consumption. According to the maximum critical safety value (400 µg Se/d) and the suggested optimal dietary intake of Se (55–200 µg Se/d) for adults [34], based on the Se concentrations in the soybean seed in the current study, which were the primary edible parts for local people, we determined the daily Se consumption for adults. Fortunately, the majority of the samples provide findings below the daily Se consumption threshold. Compared to samples treated with non-Se fertilizers, those treated with Se fertilizers and AMF are considerably more likely to produce selenium-rich agricultural products. As a result, the combination of AMF with Se fertilizer is a promising method for ongoing soybean biofortification.

## 5. Conclusions

Our study found that mycorrhizal inoculation increased soybean biomass regardless of Se fertilization and that the combined use of AMF inoculation and Se fertilization enhanced the bioavailability of Se in the soil, resulting in higher Se accumulation in soybeans. Moreover, AMF inoculation increased the proportion of organic Se in soybean seeds treated with Se fertilization. In addition, the concentration of Se in soybean seeds treated with AMF and Se fertilizer did not exceed the thresholds required to supply an adult’s daily intake of Se. According to this study, the combined use of Fm and Se fertilizers is a technique that may be utilized to improve the accumulation of organic Se in soybean seed, according to this study, which outlines some critical role of plant–fungus interactions in soil on Se biofortification. However, the impact of this approach on the uptake of Se by crops grown in field conditions remains to be investigated.

## Figures and Tables

**Table 1:** Effect of AMF inoculation and Se fertilization on dry weight of soybean individual organs and AMF colonization rates of roots ^a^.

Selenium Fertilization	Inoculation Treatment	Colonization Rate (%)	Bean (g/Pot) ^b^	Leaf and Stem (g/Pot)	Root (g/Pot)
Non-Se	NM	-	2.55 ± 1.02b	3.26 ± 1.09bc	0.26 ± 0.01b
	Fm	30.13 ± 0.81b	5.99 ± 0.25a	4.72 ± 0.36ab	0.54 ± 0.21a
	Ce	30.77 ± 1.05b	4.87 ± 0.63a	4.75 ± 0.96ab	0.57 ± 0.16a
+Se	NM	-	2.08 ± 0.23b	3.01 ± 0.13c	0.21 ± 0.03b
	Fm	34.13 ± 0.26a	5.15 ± 0.77a	5.65 ± 1.01a	0.51 ± 0.12a
	Ce	31.69 ± 1.06b	5.13 ± 0.56a	5.62 ± 0.91a	0.42 ± 0.07ab
Source of variation	Se	***	NS	NS	NS
	AMF	***	***	***	**
	Se×AMF	***	NS	NS	NS

^a^ Data presented are the means ± SE of three independent replications. ^b^ Within each column, data with different bold letters reveal significant differences from each other according to Duncan’s test (*p* ≤ 0.05). NM indicates the treatment without AMF, Ce indicates *Claroideoglomus etunicatum*, Fm indicates *Funneliformis mosseae*. NS indicates not significant at *p* > 0.05; ** at *p* ≤ 0.01; *** at *p* ≤ 0.001.

**Table 2:** Significance of main treatment effects and their interaction on Se fertilizer application and AM fungus inoculation.

Source of Variation	Se Concentration in Plant (mg/kg)			
	Bean	Pod	Stem and Leaf	Root
Se	***	***	***	***
AMF	**	***	***	**
Se × AMF	**	**	NS	NS

NS indicates not significant at *p* > 0.05; ** at *p* ≤ 0.01; *** at *p* ≤ 0.001.

**Table 3:** Effects of AMF inoculation and Se fertilizations on the bioaccumulation factor (BAF) and transport coefficient (TC_S_) of Se in soybean ^a^.

Selenium Fertilization	Inoculation Treatment	BAF _roots_	TC_roots-straws_^b^	TC_straws to pods_	TC _pods to seeds_
Non-Se	NM	0.27 ± 0.05d	0.40 ± 0.05c	0.43 ± 0.05b	0.69 ± 0.10c
	Fm	0.37 ± 0.06c	0.51 ± 0.11c	0.50 ± 0.14b	0.68 ± 0.14bc
	Ce	0.33 ± 0.01cd	0.59 ± 0.07bc	0.52 ± 0.09b	0.52 ± 0.09bc
+Se	NM	0.47 ± 0.02b	0.52 ± 0.15c	0.49 ± 0.10b	0.78 ± 0.13ab
	Fm	0.56 ± 0.02a	0.80 ± 0.09a	0.80 ± 0.14a	0.98 ± 0.09a
	Ce	0.55 ± 0.03a	0.72 ± 0.09ab	0.76 ± 0.09a	0.92 ± 0.09a
Source of variation	Se	***	**	**	***
	AMF	**	**	*	*
	Se×AMF	NS	NS	NS	NS

^a^ Data presented are the means ± SE of three independent replications. ^b^ Within each column, data with different bold letters reveal significant difference from each other according to Duncan’s test (*p* ≤ 0.05). NM indicates the treatment without AMF, Ce indicates *Claroideoglomus etunicatum*, Fm indicates *Funneliformis mosseae*. NS indicates not significant at *p* > 0.05; * at *p* ≤ 0.05; ** at *p* ≤ 0.01; *** at *p* ≤ 0.001.

**Table 4:** Daily intake of Se (μg) for adults from soybean.

Selenium Fertilization	Inoculation Treatment	Daily Intake of Se (μg)
Non-Se	NM	32.66
	Fm	43.24
	Ce	29.44
+Se	NM	65.55
	Fm	93.15
	Ce	86.25

## Data Availability

The data presented in this study are available on request from the corresponding author.
